# Awareness and Training in Medical Physics: An Avenue to Enhance Cancer Care Delivery in a Low-Resource Setting

**DOI:** 10.1200/GO.20.00036

**Published:** 2020-06-08

**Authors:** Emmanuel O. Oyekunle, Bidemi I. Akinlade, Iyobosa B. Uwadiae, Chibuzo B. Madu

**Affiliations:** ^1^ Medical Physics Unit, Department of Radiation Oncology, University College Hospital, Ibadan, Nigeria

## Abstract

**PURPOSE:**

Awareness and training in medical physics (MP) is crucial to enhance productivity and safety in cancer management. We evaluated the impact of the pioneer teaching hospital in Nigeria on awareness and training in MP in an attempt to appraise and enhance cancer care delivery.

**METHODS:**

We reviewed physics students’ training records in the MP unit of University College Hospital (UCH), Ibadan, Nigeria, from June 2009 to June 2019. These included demographic data, institutions, levels of study, time and training duration, and contact details. Trainees were contacted for updated information on their professional status. Awareness of the profession had been created at UCH and institutions within and outside Ibadan. Data were analyzed using Microsoft Excel 2016.

**RESULTS:**

One hundred thirteen trainees (mean age, 24.1 ± 2.3 years) composed of 65.5% male and 34.5% female students attended UCH within the 10-year period. They were categorized as undergraduates, (n = 83; 73.5%), Master’s students (n = 25; 22.1%), Doctoral students (n = 2; 1.8%), and workers within nuclear field (n = 3; 2.7%). Of the 83 undergraduates, 5 (6.0%) were on training as of mid-September 2019, 25 (30.1%) were still in universities or serving in the mandatory national youth service, 11 (13.3%) were in the MP profession, and 42 (50.6%) were in other career pathways. Candidates’ institutions spread across 5 of the 6 geopolitical zones in the country. Furthermore, 207 physics students took part in awareness seminars at 2 universities in commemoration of International Day of Medical Physics.

**CONCLUSION:**

Candidates from almost all parts of Nigeria benefitted from workplace experience at UCH, which continues to promote the profession. A greater feat will be attained when the University of Ibadan commences MP postgraduate programs.

## INTRODUCTION

The evolution of the medical physics (MP) profession and its awareness in Nigeria is developing at a faster rate than it was 15 years ago.^1^ MP is an applied branch of physics concerned with the application of the concepts and methods of physics to the diagnosis and treatment of human disease.^2^ In Nigeria, this career line has been faced with poor recognition and training, with a potential negative impact on cancer care delivery and health care in general. Recognition of the prospective career path by undergraduate students in physics has been considerably low in Nigeria. This could be attributed to the few clinical medical physicists available in the country. Moreover, many health professionals are unaware of the clinical role played by medical physicists because they do not often interact closely with patients. Furthermore, the relatively low volume of medical physicists in academics who could increase undergraduate students’ awareness of the career has contributed to this trend.

**CONTEXT**

**Key Objective**
Our aim was to assess the importance of awareness and training in medical physics (MP) for cancer care delivery, particularly in low-resource settings.
**Knowledge Generated**
Of physics undergraduates, 13.3% who had enrolled in workplace experience at University College Hospital, Ibadan, Nigeria, over a 10-year period chose the MP career path. Different approaches were successfully used to raise awareness of MP within and outside University College Hospital.
**Relevance**
The current study has indicated a need for hospitals that offer workplace MP experience to perform periodic appraisals with a view to assessing their contribution to MP manpower development. It would also make low- and middle-income countries aware of the importance of taking MP awareness beyond hospitals to tertiary institutions where physics students could be reached on a larger scale. In Nigeria, the passage of the outstanding Medical Physics Bill and a greater commitment of government to infrastructural development are considered urgent in the interest of enhancing cancer care delivery.

Another means of sensitization is through Students’ Industrial Work Experience Scheme (SIWES). SIWES is an acceptable training program and part of the approved academic curriculum for undergraduate degree programs in Nigerian universities.^3^ It involves the cooperation of students, universities, and employers, and exposes students to equipment and professional ethics in industries, hospitals, and other organizations. Before its establishment, industrialists and employers opined that graduates of Nigerian universities were deficient in some practical background^3^; therefore, it became a course of study that provides credit units at tertiary institutions.

The aim of the current study was to assess the impact of University College Hospital (UCH), Ibadan, Nigeria, on awareness creation and clinical training in MP within the country in the last 10 years. Nigeria is considered a lower-middle-income economy according to World Bank income classification.^4^ This work would be of interest in advancing MP, which is at a low ebb in some low- and middle-income countries (LMICs). The study was significant given that the appraisal of MP awareness and training programs at UCH helped to determine the present level of achievement and will ultimately engender enhanced productivity and safety in cancer care. Additional development of the MP profession for peaceful applications of ionization radiation in LMICs therefore is the expected end point.

## METHODS

UCH is a teaching hospital under a major university in Nigeria, the University of Ibadan.^5-7^ It is a tertiary health institution and the first to acquire nuclear medicine and high-dose-rate brachytherapy facilities in the country. The hospital has for many years been accepting students from tertiary institutions for SIWES in different disciplines. Since MP is not taught at the undergraduate level, most of the students come with little or no knowledge of the discipline.

Training components during students’ hospital work experience at our hospital often include lectures on MP topics, interactive discussions, and experimental acquisition of dosimetry data. Other aspects are acceptance testing of new radiation equipment, quality control performance, dose calculation techniques, and workplace radiation monitoring. Irrespective of training duration, candidates were usually given assignments on a regular basis, while a final assessment was normally done through written and oral tests to ensure that the program had been of intellectual value to them. Industrial training, or work-based learning, can contribute to better academic understanding in students’ final year. Work experience durations ranged from weeks to months.

The present study is based on a 10-year duration for which relevant records are available. We performed a review of candidates who were enrolled for hospital work experience in MP at UCH from June 2009 to June 2019. Records at the MP unit during SIWES candidature include demographic data, institutions and level of studies, and time and duration of training, as well as students’ contact details. Candidates were contacted to ascertain their professional status as of June 2019 for the purpose of this study. We analyzed acquired data using Microsoft Excel 2016. As part of the effort to promote the role of MP in radiation medicine in developing countries, the International Atomic Energy Agency has for several years been assisting Nigeria in the procurement of radiation equipment and manpower development through fellowship training. The agency has also been assisting LMICs through the production of materials—journals, pamphlets, etc—to enhance awareness creation of the roles of medical physicists among the appropriate stakeholders. To this end, the MP unit of UCH took the MP campaign to physics students at two notable universities in the southwestern part of the country: the University of Ibadan, Ibadan, Oyo state, and Federal University of Agriculture, Abeokuta, Ogun state. At these institutions, students were given presentations on the MP profession, its career pathway, and its significance to health care. Afterward, students were joined by some of their lecturers and engaged in interactive sessions that significantly raised their awareness about the MP career. We received feedback from some of the students, who appreciated the efforts of the MP unit and expressed a willingness to seek additional support when the need arises. Participants would expectedly be interviewed in due time through the contact details provided at the sensitization programs. The two seminars that were used to commemorate the 2019 International Day of Medical Physics (IDMP) with the theme, “It’s a Medical Physics world,” were financially supported by the Institute of Physics. In recent years, the MP unit commemorated IDMP through seminar presentations attended by various health professionals at UCH. Conducting the awareness program for students of tertiary institutions is therefore a step forward. This would be extended to other universities at intervals on the basis of approval from the respective institutional authorities. Table 1 lists the departments concerned with radiation medicine practices in the hospital and presents the related equipment in these departments. Table 2 lists the trend in the MP workforce at UCH within the review period. Whereas 4 staff members (50%) have doctoral degrees within the field, all staff have undergraduate physics backgrounds and postgraduate training in MP or radiation physics, with various levels of clinical MP proficiency.

**TABLE 1:**
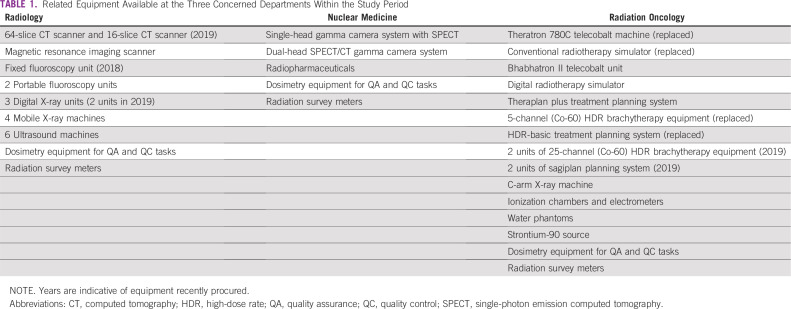
Related Equipment Available at the Three Concerned Departments Within the Study Period

**TABLE 2:**
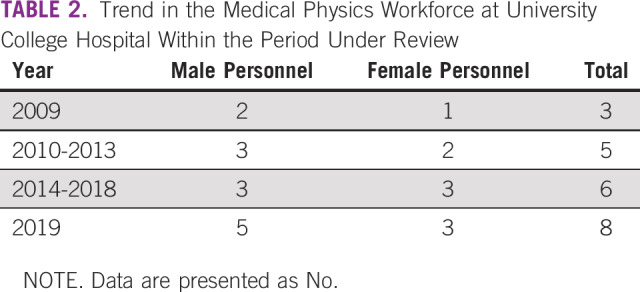
Trend in the Medical Physics Workforce at University College Hospital Within the Period Under Review

## RESULTS

**FIG 1:**
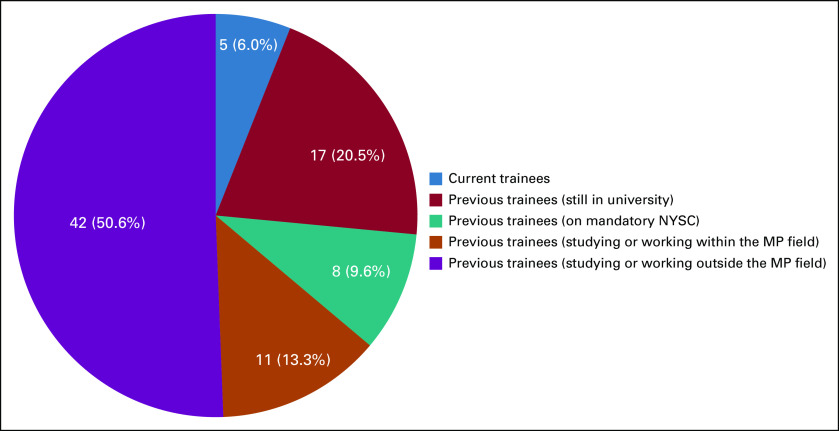
Professional status—as of mid-September 2019—of undergraduate candidates enrolled for hospital experience in medical physics (MP) at University College Hospital. NYSC, National Youth Service Corps.

A total of 113 candidates who participated in workplace experience in MP within a 10-year period at UCH were considered in this study. Table 3 lists trainees’ demographic data, including age, gender, ethnicity, marital status, and educational and academic levels, as well as the geopolitical zones of their institutions. Trainees at the undergraduate level constitute the largest group at 73.5%. Table 3 also reveals that the proportion of female students is slightly higher than one third (34.5%) of the candidates under review. Institutions that provided the trainees cut across 5 of the 6 geopolitical zones in the country (Table 3). Table 4 gives the proportion of candidates across the 10-year duration. In most cases, the MP unit of UCH enrolled 10 or more students for industrial training annually. Additional data regarding training durations of the students are lists in Table 5. Table 6 highlights approaches to MP awareness during IDMP’s commemoration at the institution. Classifications of undergraduate participants on the basis of professional status as of mid-September 2019 are shown in Figure 1. Eleven candidates representing 13.3% of the undergraduate students were found to be engaged in the MP profession after workplace experience gained at UCH. A total of 207 students were successfully reached through the awareness programs organized by the MP units at the University of Ibadan (n = 92) and Federal University of Agriculture (n = 115) in commemoration of 2019 IDMP.

**TABLE 3:**
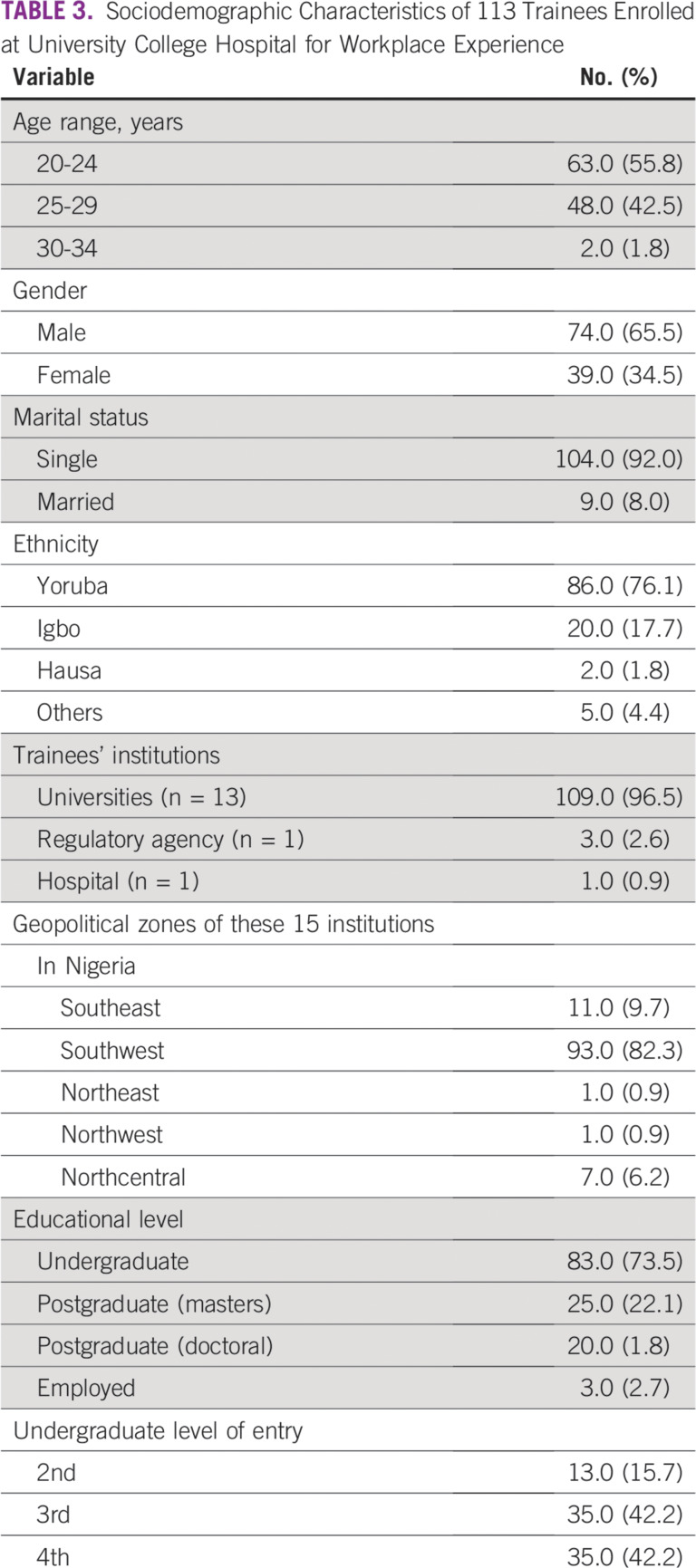
Sociodemographic Characteristics of 113 Trainees Enrolled at University College Hospital for Workplace Experience

**TABLE 4:**
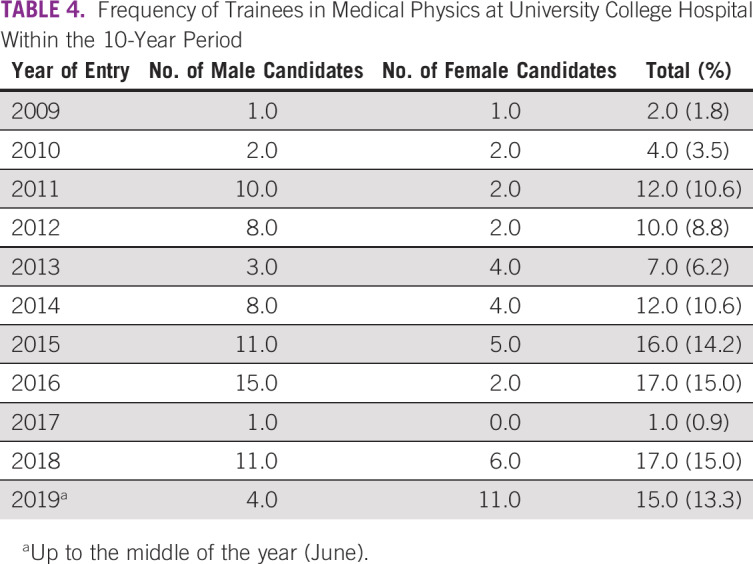
Frequency of Trainees in Medical Physics at University College Hospital Within the 10-Year Period

**TABLE 5:**
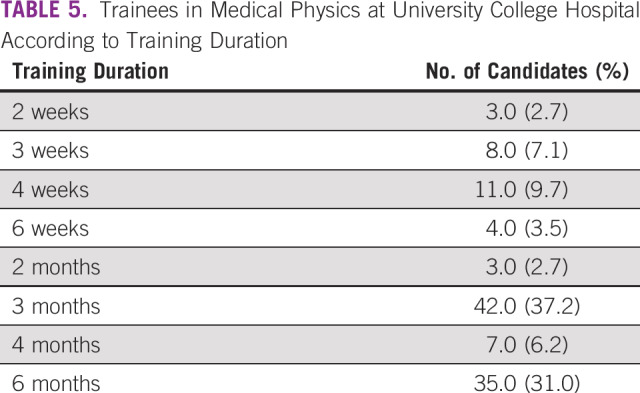
Trainees in Medical Physics at University College Hospital According to Training Duration

**TABLE 6:**
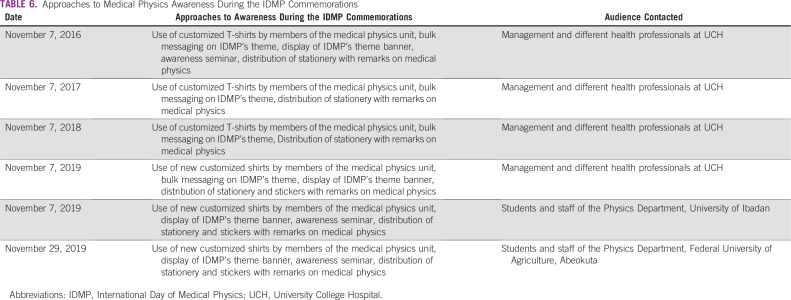
Approaches to Medical Physics Awareness During the IDMP Commemorations

## DISCUSSION

Globally, the significance of medical physicists to radiation medicine with respect to the accurate diagnosis and treatment of cancer cannot be overemphasized. Awareness creation and training in MP, particularly in resource-constrained nations like Nigeria and some LMICs, is therefore of high importance. A masters’ degree program in MP inclusive of clinical experience in a hospital is indispensable to producing the manpower needed to overcome the present dearth of clinical medical physicists required to enhance productivity in cancer care. More than one half (55.8%) of the students who had MP training experience at our hospital were younger than age 25 years (Table 3). This means that more than 70% of trainees were undergraduates. Our study further revealed that the female-to-male ratio of trainees was almost 1:2 (Table 3). We observed within the 10-year period a steady increase in the number of female physics students participating in the training scheme (SIWES), except for 2016 and 2017 (Table 4). A previous global-based study,^8^ which focused on female representation in MP manpower showed that certain LMICs had only women medical physicists or a high percentage of women MPs, whereas high-income countries reported a much lower proportion of women medical physicists than initially anticipated. On the basis of data from 66 countries, the reported proportion of women, according to the International Organization for Medical Physics, was 28%. The gender proportion obtained in our study is therefore encouraging as it could foster engagement of more women in the MP profession in Nigeria. A recent study^9^ that gathered data on the global MP workforce opined that a more inclusive environment and culture for women medical physicists would facilitate their involvement for the benefit of our profession. University students are often the dominant proportion of those who obtain hospital experience in MP (Table 3). The spread of institutions across different parts of the country highlights the recognition of UCH as an important resource center for workplace experience. In addition to undergraduate students, approximately one quarter (23.9%) of the study population had some practical experience in the field before completing their postgraduate programs. Moreover, employees of governmental institutions were inclusive in industrial attachment to our hospital (Table 3). There was a need for them to acquire additional experience as part of their continuous professional development. In 2012, UCH was involved as a resource center for high-dose–rate brachytherapy training of a Nigerian medical physicist through the International Atomic Energy Agency fellowship.

Having 5 classifications, Figure 1 shows the professional status of 83 candidates who attended UCH for hospital experience during their undergraduate studies. Of the 15 accepted for industrial attachment between January and June 2019, 5 were in the program as of mid-September 2019. The question whether academic degree programs should continue to incorporate industrial training into their curriculum is no longer a debate as the monumental benefits the nation stands to gain cannot be overemphasized. Giving undergraduates the opportunity to gain some practical experience in the work environment will enable them to apply the theoretical knowledge acquired in earlier years at the university to related working sites.^10^ The authors, through interaction with some previous physics students, observed that those who had workplace experience at UCH performed better in their final year in school, as they had a clear sense of direction in their projects. This is one of the objectives of industrial training that is work-based learning.^11^

Seventeen students who gained work experience at UCH between 2018 and 2019 are in the process of completing their undergraduate degrees. Eight students who graduated earlier are presently serving in the mandatory National Youth Service Corps. The National Youth Service Corps is a scheme set up by the Nigerian government to involve graduates in national development.^12^ The program deploys new graduates of tertiary institutions for a 1-year period to other parts of the country that they might otherwise not have been familiar with. It provides a means for fostering national unity^13^ and is therefore known as a national service year.^12^

Of most importance, our study revealed that 13.3% of undergraduate students enrolled for work experience at UCH had chosen the MP career path. This interest group consists of candidates presently working, studying, or those who have already concluded postgraduate programs in MP. One student completed a doctoral degree program at a university outside Ibadan and is presently working as a clinical medical physicist. Industrial training continues to offer a symbiosis between students and the hospital in a win-win situation. As the students acquire needed experience, the hospital also benefits from their supervised services. Graduates who gained work experience revealed that this exposure was the most important link between their theoretical knowledge and the practical situation of entering the workforce. This fact was corroborated by Fry et al,^14^ as well as by Hess and Kelly.^15^ Trotskovy and Sabag^16^ posited that students must also have the opportunity to identify differences in the “traditional learning process in the academic environment and real-design process in the industrial environment.” The authors opine that the estimated proportion of this particular group (13.3%) would reasonably increase in the near future. This is based on the fact that 36.1% of undergraduates under review are yet to choose a career path.

The final group of those studying or working outside MP represents approximately one half (50.6%) of undergraduate trainees. That some in this category may later embrace the MP profession cannot be ruled out. Our findings revealed that at least one quarter of candidates in this group are engaged as college teachers or are working within the telecommunications, information technology, and banking industries. Financial constraint was identified as a major factor preventing the continuation of their education at the postgraduate level. A few candidates also attributed a lack of interest in the MP profession to the existing poor remuneration and recognition in the country. Therefore, there is the possibility that more candidates may further their education in MP when there the aforementioned circumstances change. Other hospitals in the country with relevant facilities also receive physics students at tertiary institutions for workplace experience; however, at the time of this study, there were no published data for comparison with the data in Figure 1. Our institution has also taken the lead in awareness creation within the hospital, being the first to formerly celebrate IDMP in Nigeria in 2016 when the 4th commemoration was internationally observed. Highlights of the awareness efforts during commemorations of IDMP by the MP unit from 2016 to 2019 are listed in Table 6. The recent awareness outreach to university physics students as part of the IDMP commemoration was also a new initiative in Nigeria undertaken by the MP unit at UCH. There is thus a need for other hospitals to offer workplace physics experience to perform a periodic assessment and create appreciable awareness of the profession, particularly during IDMP, to contribute to MP manpower development. This study is also expected to inform LMIC institutions about the importance of taking awareness beyond the hospitals to tertiary institutions where physics students could gain career orientation on a larger scale.

In Nigeria, with a population of more than 200 million as of 2019,^17^ there are few clinical medical physicists to attend to the numerous diagnostic centers and the gradually increasing number of radiation oncology centers in the country. Tsapaki et al^9^ previously reported a total number of 100 medical physicists in Nigeria, few of whom are in clinics. The trend in the MP workforce at UCH over a 10-year period, presented in Table 2, points to this effect. As of 2019, there are 8 clinical medical physicists serving the 3 departments—radiology, radiation oncology, and nuclear medicine—concerned with radiation medicine. More qualified clinical medical physicists are required in the country for efficient and effective radiation practices. A bill to establish the Nigerian College of Medical Physics as the regulatory body for the practice of the profession and to standardize the clinical residency program is already in the senate undergoing the process of passage into law, albeit slowly.^18^ The MP unit of UCH continues to contribute to the development of the profession in Nigeria in a bid to address the shortfall of human capital. The unit is presently faced with a low staff-to-student ratio. This and other challenges, such as inadequate workspace and limited equipment, often limit the number of students enrolled in the workplace experience program. Greater commitment of government toward infrastructure development is urgently needed to ensure that the 8 teaching hospitals in the country presently with radiotherapy machines^19^ are adequately equipped while *s*etting up additional facilities.

To conclude, this study reports the impact of workplace experience and awareness provided by the pioneer teaching hospital in Nigeria on the development of MP in the country. Work experience provided at UCH for students with a keen interest in the application of physics to medicine continues to offer hope for the advancement of the MP profession. The MP unit of UCH underscores the need for increased awareness of the profession in the country and has taken the lead. This study has indicated a strong need for a continuous attachment of interested physics students to UCH and, by extension, to other relevant hospitals. It could serve as a model for other training and awareness programs and thereby support the advocacy for the profession. Steps taken in this direction would engender improved productivity in MP aspects of radiation medicine, particularly in LMICs. It is expedient that the MP bill before the Nigerian senate is given speedy passage to further accentuate the overall development of the profession.
